# The complete chloroplast genome sequences of four *Viola* species (Violaceae) and comparative analyses with its congeneric species

**DOI:** 10.1371/journal.pone.0214162

**Published:** 2019-03-20

**Authors:** Kyeong-Sik Cheon, Kyung-Ah Kim, Myounghai Kwak, Byoungyoon Lee, Ki-Oug Yoo

**Affiliations:** 1 Department of Biological Science, Sangji University, Wonju, Gangwon, South Korea; 2 Department of Biological Sciences, Kangwon National University, Chuncheon, Gangwon, South Korea; 3 Plant Resources Division, National Institute of Biological Resources, Incheon, South Korea; National Cheng Kung University, TAIWAN

## Abstract

We report the complete chloroplast genomes of four *Viola* species (*V*. *mirabilis*, *V*. *phalacrocarpa*, *V*. *raddeana*, and *V*. *websteri*) and the results of a comparative analysis between these species and the published plastid genome of the congeneric species *V*. *seoulensis*. The total genome length of the five *Viola* species, including the four species analyzed in this study and the species analyzed in the previous study, ranged from 156,507 (*V*. *seoulensis*) to 158,162 bp (*V*. *mirabilis*). The overall GC contents of the genomes were almost identical (36.2–36.3%). The five *Viola* plastomes each contained 111 unique genes comprising 77 protein-coding genes, 30 transfer RNA (tRNA) genes, and 4 ribosomal RNA (rRNA) genes. Among the annotated genes, 16 contained one or two introns. Based on the results of a chloroplast genome structure comparison using MAUVE, all five *Viola* plastomes were almost identical. Additionally, the large single copy (LSC), inverted repeat (IR), and small single copy (SSC) junction regions were conserved among the *Viola* species. A total of 259 exon, intron, and intergenic spacer (IGS) fragments were compared to verify the divergence hotspot regions. The nucleotide diversity (Pi) values ranged from 0 to 0.7544. The IR region was relatively more conserved than the LSC and SSC regions. The Pi values in ten noncoding regions were relatively high (>0.03). Among these regions, all but *rps19*-*trnH*, *petG*-*trnW*, *rpl16*-*rps3*, and *rpl2*-*rpl23* represent useful molecular markers for phylogenetic studies and will be helpful to resolve the phylogenetic relationships of *Viola*. The phylogenetic tree, which used 76 protein-coding genes from 21 Malpighiales species and one outgroup species (*Averrhoa carambola*), revealed that Malpighiales is divided into five clades at the family level: Erythroxylaceae, Chrysobalanaceae, Euphorbiaceae, Salicaceae, and Violaceae. Additionally, Violaceae was monophyletic, with a bootstrap value of 100% and was divided into two subclades.

## Introduction

With the development of next-generation sequencing (NGS) technology, many studies have performed whole chloroplast genome sequencing. These studies have provided much information about plant taxonomy and evolution. The rapidly evolving loci identified by these studies are very important for resolving unclear phylogenetic relationships because they have a higher resolving power than that of traditional molecular markers. Therefore, many studies have focused on finding genic regions among specific families or genera to provide useful information about molecular markers for further studies [1–6].

Violaceae Batch. consists of approximately 22 genera and 1000–1100 species of herbs, shrubs, lianas, and trees [7–9]. The genus *Viola* L. comprises 583–620 species and is distributed mainly in temperate and tropical regions [7–8,10–11]. This genus is known as one of more difficult groups to classify because of the very similar external morphology characters among species and the many intermediate forms that exist due to frequent interspecies hybridization between closely related species [12–15].

For this reason, although many studies have been carried out, the phylogenetic relationships of *Viola* are still unclear among sections and/or species [7, 11, 13, 16–19]. This lack of clarity is because the molecular markers used by previous studies has low resolution to evaluate the phylogenetic relationships of *Viola*. Therefore, to correctly evaluate the phylogenetic relationships of *Viola*, the most suitable molecular markers should be selected via analyses of the sequence variation at each locus.

Among the four *Viola* species discussed in this study, three species (*V*. *mirabilis* L., *V*. *raddeana* Regel and *V*. *websteri* Hemsl.) are very rare because they are endangered in Korea. In particular, it is urgent to establish a conservation strategy for *V*. *raddeana* because this species only has a single, relatively small population of individuals in Gyeongsangnam-do Province [20]. *V*. *phalacrocarpa* Maxim. is not an endangered species, but various taxonomic data are needed because its taxonomic level is ambiguous due to its close relationship to *V*. *seoulensis*.

Here, we report the whole chloroplast genome sequences of four *Viola* species (*V*. *mirabilis*, *V*. *phalacrocarpa*, *V*. *raddeana*, and *V*. *websteri*) and the results of a comparative analysis between these species and the published genome of a congeneric species (*V*. *seoulensis* Nakai). The main goal of this study was to provide important information about the most suitable chloroplast molecular markers for further studies to solve unclear phylogenetic relationships of *Viola* via the calculation of the rate of evolution of each chloroplast genome loci. Furthermore, this study could expand the current understanding of the chloroplast genome characteristics of the genus *Viola* and provide basic chloroplast phylogenomic data for Violaceae, thus supporting the development of conservation strategies for endangered Violaceae species.

## Materials and methods

### Sample collection, DNA extraction

Among the four *Viola* species in this study, three (*V*. *mirabilis*, *V*. *raddeana*, and *V*. *websteri*) are legally protected species. Therefore, samples of these species were collected with permission from the Ministry of Environment in Korea, with the following license numbers: 2015–15 (*V*. *raddeana*) and 2015–39 (*V*. *mirabilis* and *V*. *websteri*).

Fresh leaf materials of individual *V*. *mirabilis*, *V*. *raddeana*, *V*. *phalacrocarpa*, and *V*. *websteri* were collected from Hutan-ri in Gangwon-do Province (37°11'09"N 128°22'16"E), Youngdang-ri in Gyeongsangnam-do Province (35°22'15"N 128°54'34"E), Mt. Oeum in Gangwon-do Province (37°35'53"N 127°57'15"E), and Bugok-ri in Gangwon-do province (37°19'36"N 128°03'15"E) in South Korea, respectively. The voucher specimens were deposited in the National Institute of Biological Resources Herbarium (KB) and the Kangwon National University Herbarium (KWNU). The voucher numbers are GEIBVP0000373630 (*V*. *mirabilis*), NIBRVP0000454691 (*V*. *raddeana*), KWNU91089 (*V*. *phalacrocarpa*), and GEIBVP0000373612 (*V*. *websteri*). Total DNA was extracted from approximately 100 mg of fresh leaves using a DNA plant mini kit (Qiagen Inc., Valencia, CA, USA).

### Sequencing, assembly, annotation, genome comparison and repeat analysis

Genomic DNA was used for sequencing by an Illumina MiSeq (Illumina Inc., San Diego, CA, USA) platform. The DNA of *Viola* species were sequenced to produce 8,920,660–9,244,544 raw reads with a length of 301 bp. These reads were aligned with the reference genome of *Viola seoulensis* (GenBank accession number: KP749924). A total 542,183 to 667,526 reads were mapped to the reference genome. The genome coverage was estimated using CLC Genomics Workbench v7.0.4 software (CLC-bio, Aarhus, Denmark). The genome coverages of the sequencing data from *V*. *mirabilis*, *V*. *phalacrocarpa*, *V*. *raddeana*, and *V*. *websteri* were 1002, 875, 986, and 1073, respectively.

The protein-coding genes, transfer RNAs (tRNAs), and ribosomal RNAs (rRNAs) in the plastid genome were predicted and annotated using Dual Organellar GenoMe Annotator (DOGMA) with the default parameters [21] and manually edited by a comparison with the published chloroplast genome sequence of Violaceae. tRNAs were confirmed using tRNAscan-SE [22]. The circular plastid genome map was drawn using OGDRAW [23].

The complete chloroplast genomes of five *Viola* species, including the four *Viola* species of this study and the previously published species (*V*. *seoulensis*), were compared using MAUVE [24]. The large single copy/inverted repeat (LSC/IR) and inverted repeat/small single copy (IR/SSC) boundaries of these species were also compared and analyzed.

The REPuter program [25] was used to identify repeats (forward, reverse, palindrome, and complement sequences). The size and identity of the repeats were limited to no less than 30 bp and 90%, respectively. The simple sequence repeats (SSRs) in the chloroplast genome of the five *Viola* species were detected using Phobos v.3.3.12 (http://www.ruhr-uni-bochum.de/ecoevo/cm/cm_phobos.htm). Repeats were ≥10 bp in length and had three repeat units for mono-, di- tri-, tetra-, penta- and hexanucleotides.

### Divergence hotspot identification

The five chloroplast genomes of *Viola* were analyzed to identify rapidly evolving molecular markers that can be used in further phylogenetic studies of *Viola*. Both coding and noncoding region fragments in each plastid genome were extracted separately by applying the “Extract” option of Geneious v7.1.8 (Biomatters Ltd., Auckland, New Zealand). Then, the homologous loci were aligned individually using MAFFT [26]. To analyze nucleotide diversity (Pi), the total number of mutations (Eta), average number of nucleotide differences (K) and parsimony informative characters (PICs) were determined using DnaSP [27].

### Phylogenetic analyses

A total of 76 protein-coding genes from 22 species were compiled into a single file of 83,600 bp and aligned with MAFFT [26]. Twenty-one Malpighiales were selected as the ingroups, and one species from Oxalidaceae R. Br. (*Averrhoa carambola* L.) was chosen as the outgroup (S1 Table). Maximum likelihood (ML) analyses were performed using RAxML v7.4.2 with 1000 bootstrap replicates and the GTR+I model [28]. Bayesian inference (ngen = 1,000,000, samplefreq = 200, burninfrac = 0.25) was carried out using MrBayes v3.0b3 [29], and the best substitution model (GTR+I) was determined by the Akaike information criterion (AIC) in jModeltest version 2.1.10 [30].

## Results

### Chloroplast genome features of five *Viola* species

**Fig 1:**
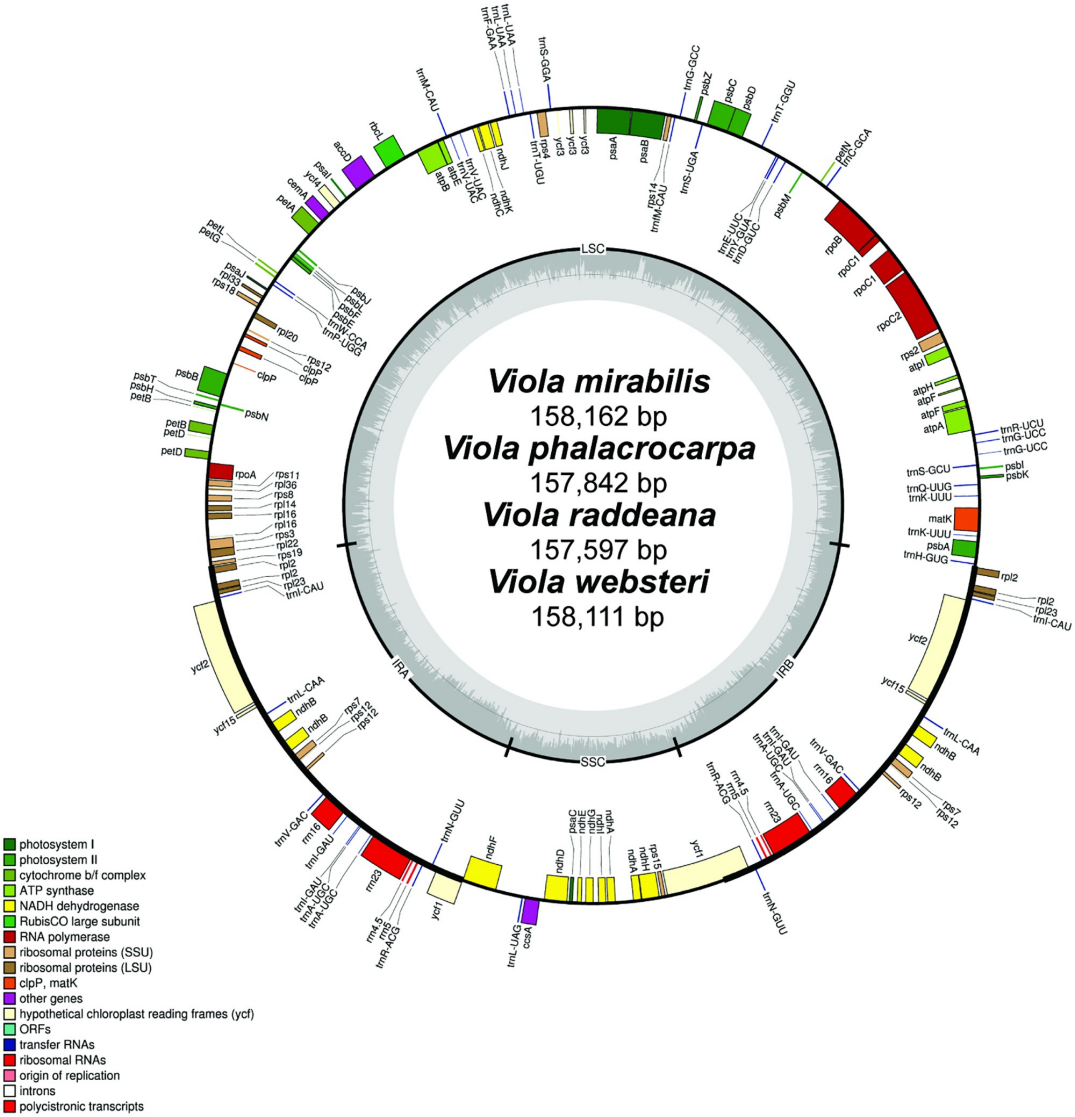
Map of the chloroplast genome of four *Viola* species. Genes inside the circle are transcribed clockwise, gene outside are transcribed counter-clockwise. The dark gray inner circle corresponds to the GC content, the light-gray to the AT content.

The chloroplast genomes of *V*. *mirabilis* (accession no. MH229816), *V*. *phalacrocarpa* (accession no. MH229817), *V*. *raddeana* (accession no. MH229818), and *V*. *websteri* (accession no. MH229819) have been submitted to GenBank of National Center for Biotechnology Infromation (NCBI). The total length of the chloroplast genomes of the five *Viola* species, i.e., the four species analyzed in this study and species analyzed in a previous study (*V*. *seoulensis*), ranged from 156,507 (*V*. *seoulensis*) to 158,111 bp (*V*. *websteri*). All five *Viola* plastid genomes exhibited the typical quadripartite structure, consisting of a pair of IR regions (26,404–27,166 bp) separated by an LSC region (85,691–86,588 bp) and an SSC region (17,191–18,008 bp). Their overall GC contents were almost identical (36.2–36.3%). The chloroplast genomes of the five species contained 111 unique genes comprising 77 protein-coding genes, 30 tRNA genes, and 4 rRNA genes (Table 1 and Fig 1). Among the annotated genes, 14 genes (*ndhA*, *ndhB*, *petB*, *petD*, *rpl2*, *rpl16*, *rpoC1*, *rps12*, *trnK-UUU*, *trnG-UCC*, *trnL-UAA*, *trnV-UAC*, *trnI-GAU*, *trnA-UGC*) contained one intron each, and two genes (*ycf3*, *clpP*) contained two introns each.

**Table 1:** Comparison of chloroplast genome feature of five *Viola* species.

Feature	*V*. *mirabilis*	*V*. *phalacrocarpa*	*V*. *raddeana*	*V*. *seoulensis*	*V*. *websteri*
Genome size	158,162	157,842	157,597	156,507	158,111
LSC	86,565	86,367	86,460	85,691	86,588
SSC	17,351	17,305	17,289	18,008	17,191
IR	27,123	27,085	26,924	26,404	27,166
GC content	36.2	36.3	36.2	36.3	36.2
LSC	33.8	33.9	33.8	33.8	33.7
SSC	29.9	29.8	29.9	29.6	29.9
IR	42.2	42.2	42.3	42.6	42.1
Number of genes	111	111	111	111	111
Protein coding genes	77	77	77	77	77
tRNA genes	30	30	30	30	30
rRNA genes	4	4	4	4	4

**Fig 2:**
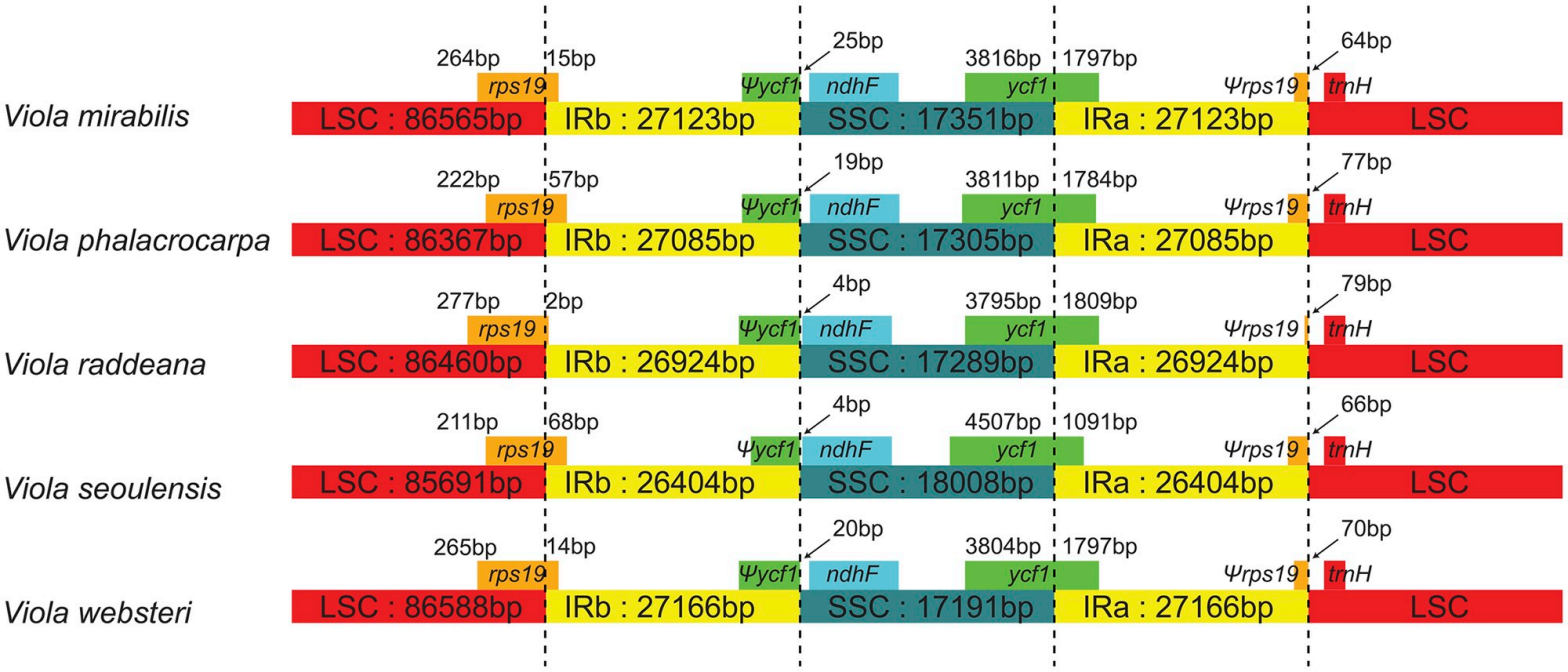
Comparison of the LSC, IR, and SSC junction positions in the five *Viola* chloroplast genomes.

The result of the chloroplast genome structure comparison using MAUVE [24] showed that all five *Viola* plastomes were the same (S1 Fig). The LSC/IR and IR/SSC boundaries were conserved in *Viola*. In all five *Viola* chloroplast genomes, *trnH-GUG* was located in the LSC near the IRa/LSC border, and *ndhF* was located in the SSC near the IRb/SSC border. Additionally, pseudogenes of *rps16* and *ycf1* situated in the IRb were created by IR extending into the LSC and SSC regions, respectively (Fig 2).

**Fig 3:**
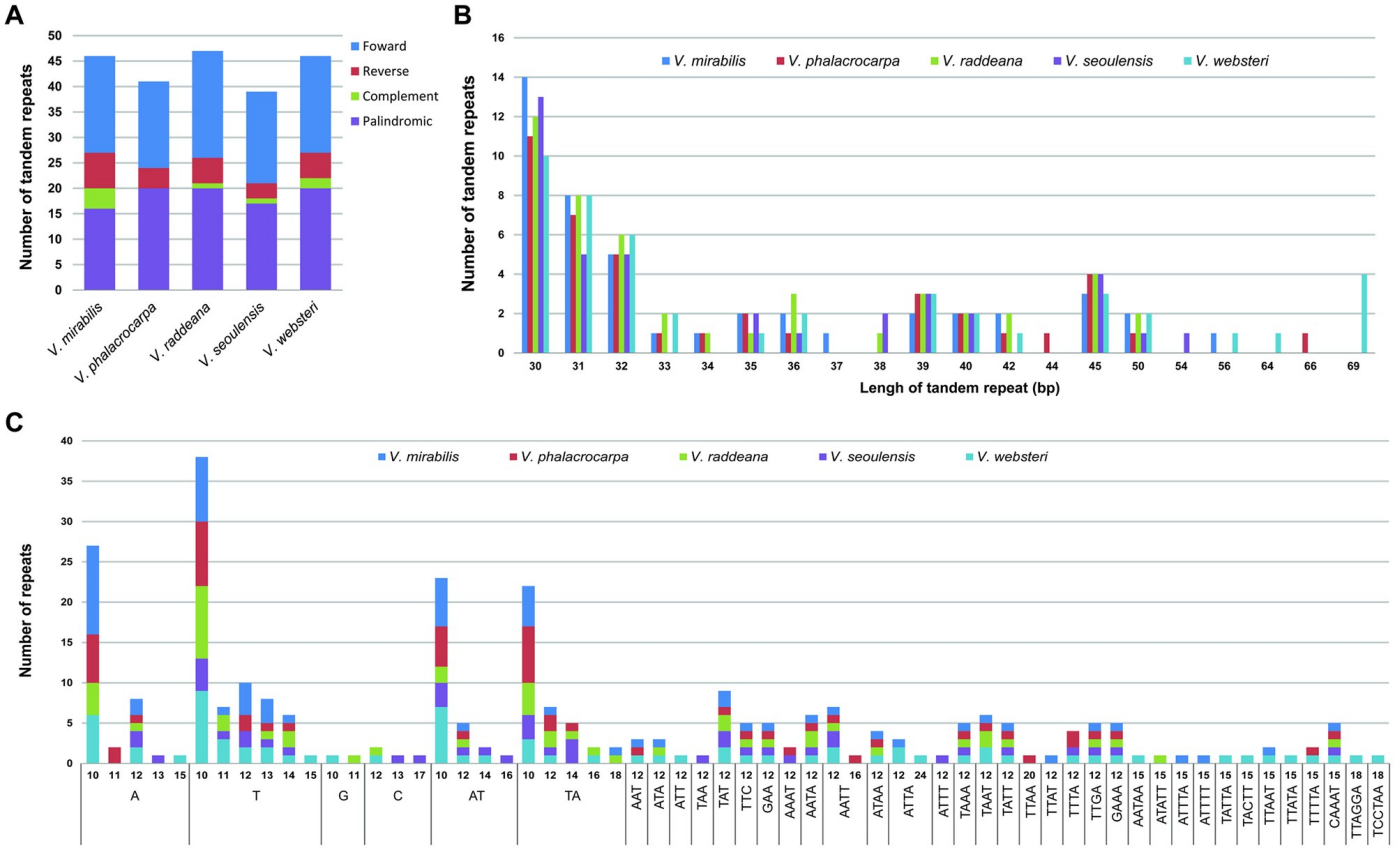
Analyses of repeated sequences in the five *Viola* chloroplast genomes. (A) Types and number of tandem repeats in five *Viola* chloroplast genomes, (B) Frequency by length of tandem repeats in five *Viola* chloroplast genomes, (C) Frequency by type of SSRs in five *Viola* chloroplast genomes.

Four classes of tandem repeats (forward, reverse, complement and palindrome) were investigated. The number of tandem repeats for each class is shown in Fig 3A. Additionally, the tandem repeats that ranged from 30 to 39 bp were the most abundant, followed by those that ranged from 40 to 49 bp. Moreover, among the chloroplast genomes of all five *Viola* species, that of *V*. *websteri* had the highest number of tandem repeats longer than 50 bp (Fig 3B).

The analysis of SSRs indicated that six categories of SSRs, i.e., mono-, di-, tri-, tetra-, penta- and hexanucleotide, were detected. The total number of SSRs was 64 in *V*. *mirabilis*, 56 in *V*. *phalacrocarpa*, 50 in *V*. *raddeana*, 43 in *V*. *seoulensis*, and 73 in *V*. *websteri*. The most dominant of SSRs were A/T mononucleotides. Only the *V*. *websteri* chloroplast genome had all six types of SSRs, and those of the other species had five types of SSRs, excluding the hexanucleotide SSR (Fig 3C).

### Divergence hotspot regions in *Viola*

**Fig 4:**
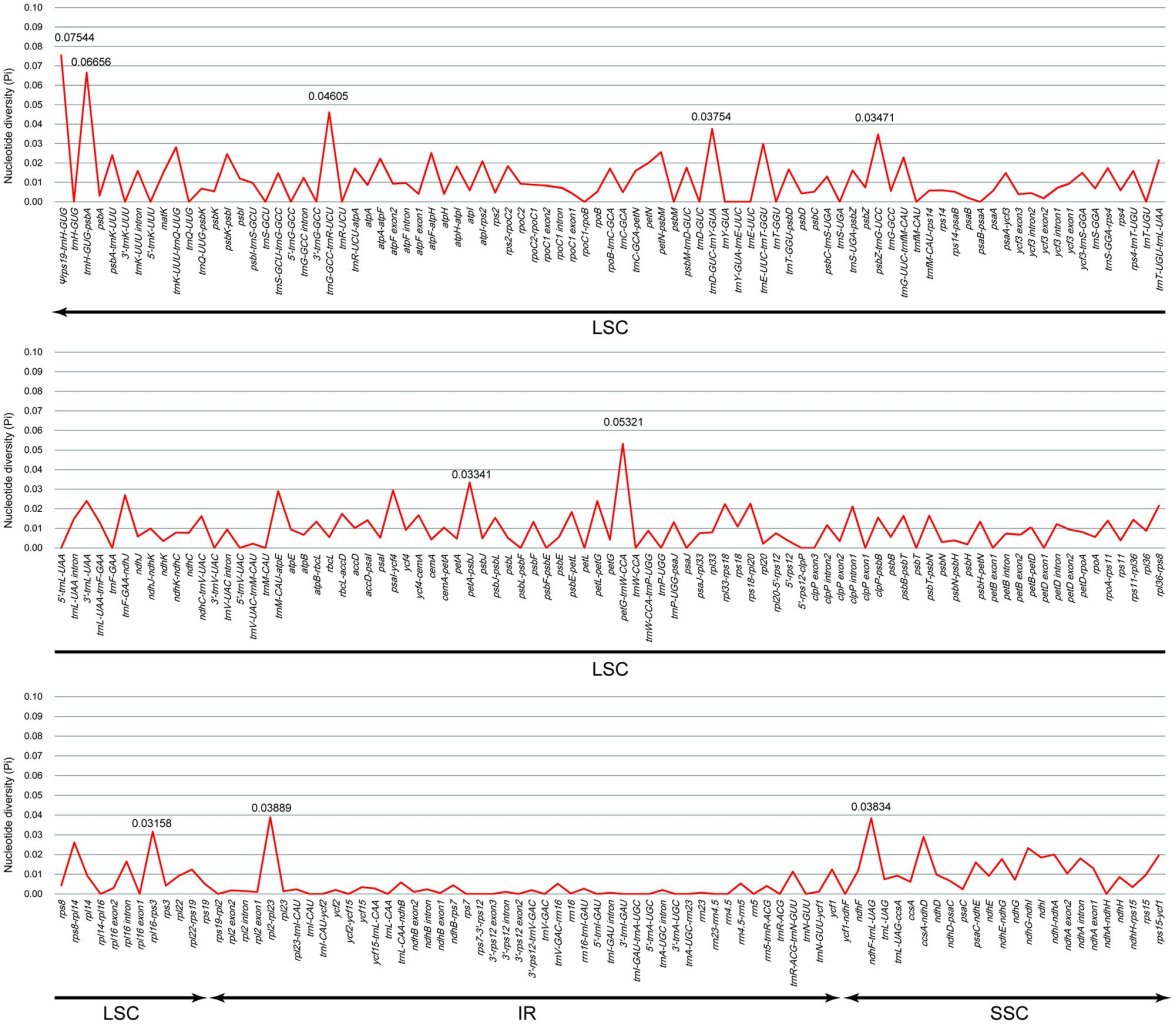
Comparison of the nucleotide diversity (Pi) values in five *Viola* species.

A total of 259 exon, intron and intergenic spacer (IGS) fragments were compared among the five *Viola* species to verify divergence hotspot regions. The Pi values ranged from 0 to 0.7544 (Fig 4 and S2 Table). The IR region was much more conserved than the LSC and SSC regions because the IR region had the most fragments with a relatively low Pi value. The Pi value was 0.03 or more in ten regions. Among these, eight (*rps19*-*trnH*, *trnH*-*psbA*, *trnG*-*trnR*, *trnD*-*trnY*, *psbZ*-*trnG*, *petA*-*psbJ*, *petG*-*trnW*, *rpl16*-*rps3*) were located in the LSC region, one (*rpl2*-*rpl23*) was located in the IR region, and one (*ndhF*-*trnL*) was located in the SSC region. Additionally, all regions with a high Pi value were IGSs.

### Phylogenetic relationships of Violaceae within Malpighiales

**Fig 5:**
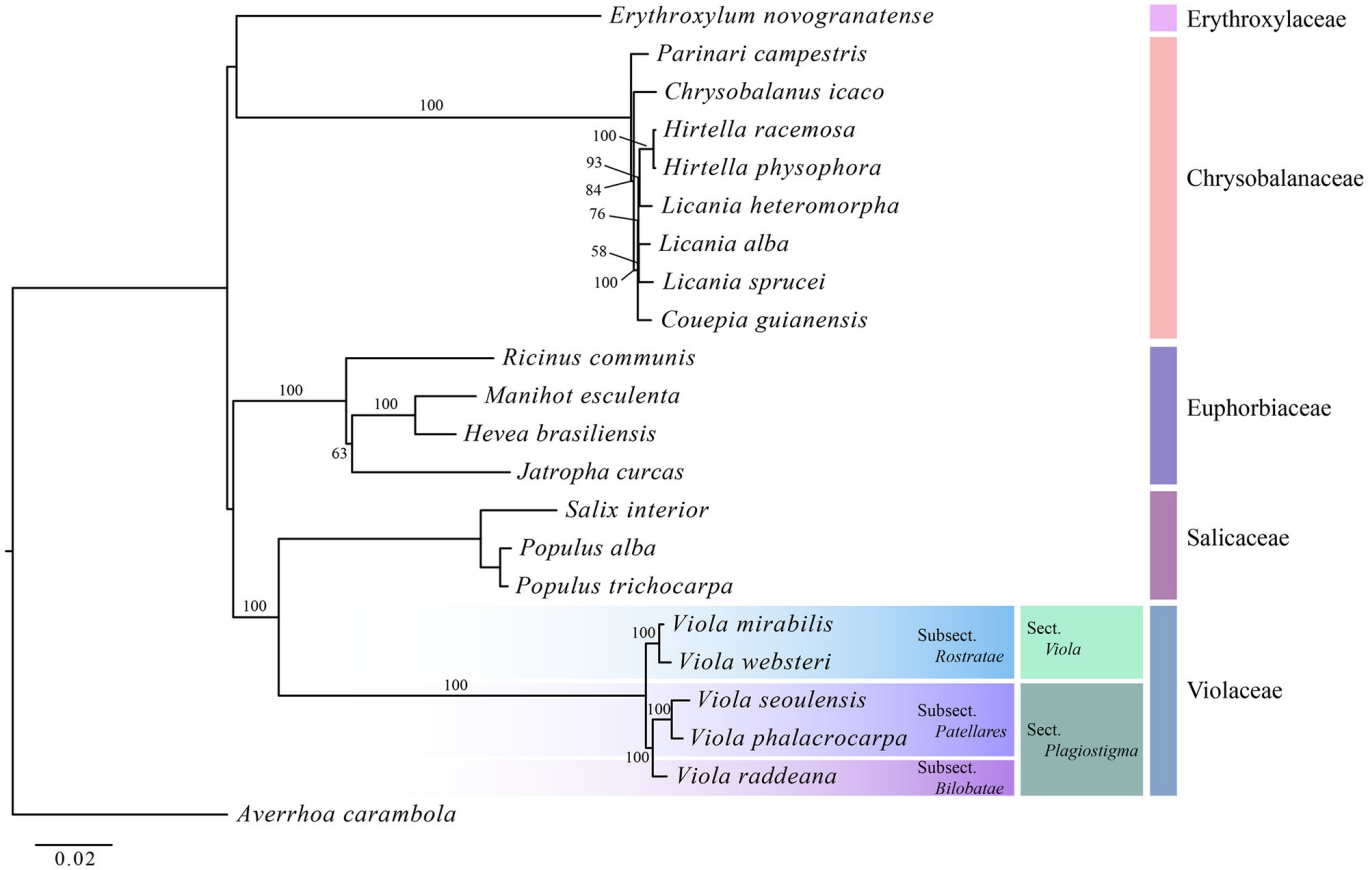
The phylogenetic tree based on 76 protein coding genes from 22 species of Malpighiales and one outgroup.

The ML tree using 76 protein-coding genes clearly divided into five clades at the family level: Erythroxylaceae Kunth, Chrysobalanaceae R. Br., Euphorbiaceae Juss., Salicaceae Mirb., and Violaceae. Violaceae and *Viola* were monophyletic with a bootstrap value of 100% and a sister to Salicaceae. Additionally, Violaceae was divided into two subclades: sect. *Viola* W. Becker (subsect. *Rostratae* W. Becker) and sect.*Plagiostigma* Solid (subsect. *Patellares* (Boiss.) Rouy & Foucaud and *Bilobatae* (W. Becker) W. Becker) (Fig 5).

## Discussion

### Comparison of the chloroplast genomes of five *Viola* plastomes

**Fig 6:**
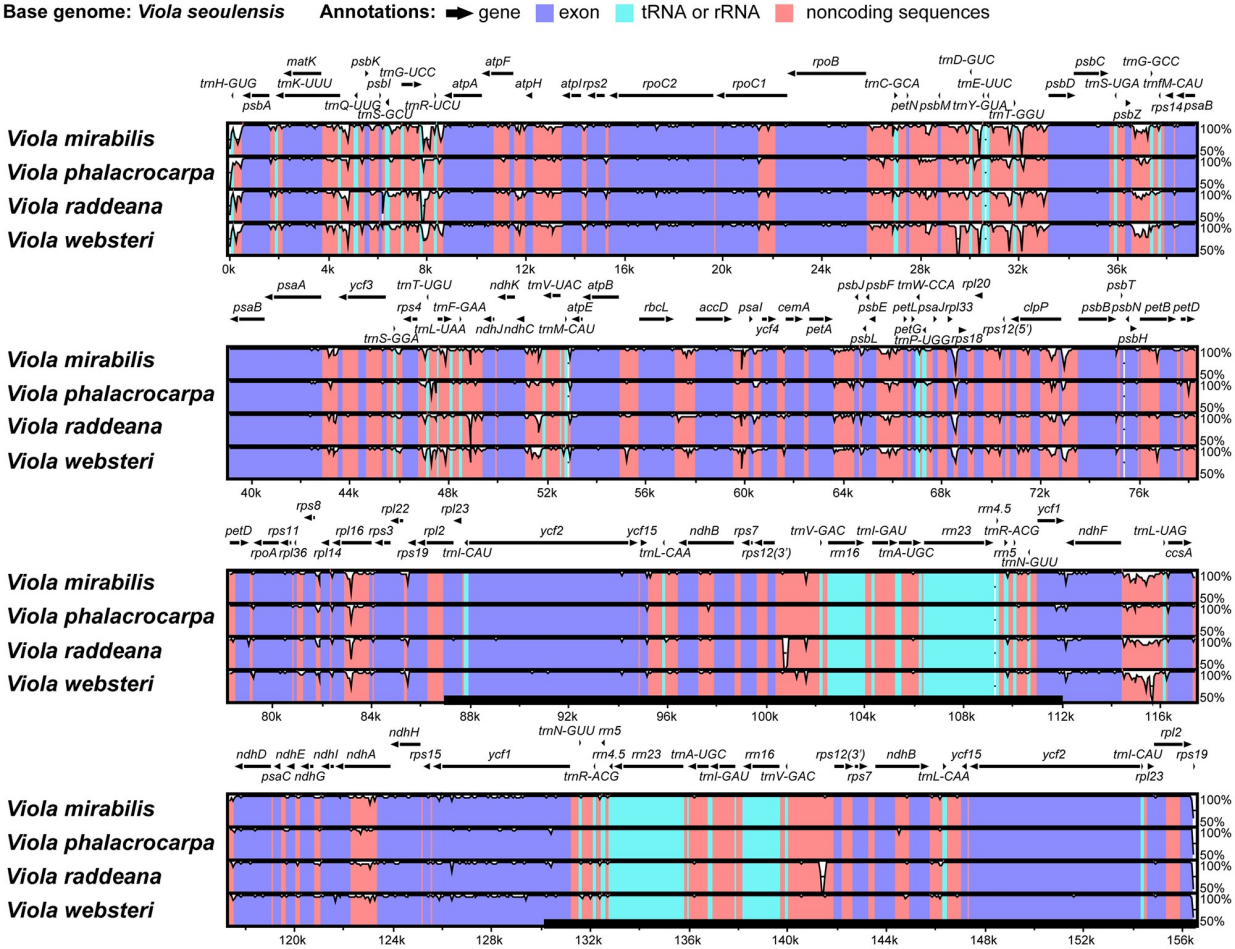
Sequence alignment of five *Viola* plastid genomes in mVISTA.

Many recent studies have been carried out to solve taxonomic problems between related taxa using complete chloroplast genome sequences. The chloroplast genome is known to be very conservative in land plants, but structural changes in chloroplast genomes, such as gene duplication and deletion and inversion due to occasional rearrangements, provide important taxonomic data [31–37]. This study showed that the gene order of five *Viola* chloroplast genomes was identical, and the sequence identity was also very similar among species in most of the chloroplast regions (Fig 6 and S1 Fig). Therefore, these results indicate that the plastid genome of *Viola* is very conservative.

*Ycf15* in *V*. *mirabilis* was 66 bp shorter than that in the other four *Viola* species because there was a premature stop codon due to a point mutation of one nucleotide (S2 Fig). This important data supports the taxonomic position of *V*. *mirabilis*. In addition, future studies should be performed to determine whether *ycf15* is a pseudogene.

The number of the tandem repeats in the five *Viola* plastomes ranged from 39 (*V*. *seoulensis*) to 47 (*V*. *raddeana*), and the number of tandem repeats according to type and length showed a slightly difference across each species (Fig 3A, 3B). The presence and abundance of repetitive sequences in the chloroplast or nuclear genome are likely to involve many phylogenetic signals [38–40]. Therefore, the different abundances of tandem repeats among the plastid genomes of the five *Viola* species may provide additional evolutionary information. In addition, the SSRs identified in this study may provide various markers for population genetic studies of *Viola* species.

### Selection of useful molecular marker regions for additional phylogenetic studies

The genus *Viola* is known as one of more difficult groups to study taxonomically since *Viola* has many morphologically similar species, and the creation of intermediate forms due to interspecific hybridization occurs freely [12–15]. Because of this external morphological complexity among species, although many taxonomic studies [7, 9, 11, 13, 16, 18] have been conducted, the taxonomic positions and phylogenetic relationships within sections level of *Viola* are remain insufficiently resolved.

The molecular markers used in previous studies, except for the ITS region of nuclear DNA, were ten chloroplast DNA sequences, *trnL*, *trnL-trnF*, *rbcL*, *atpB-rbcL*, *atpF-atpH*, *matK*, *psbA-trnH*, *psbK-psbI*, *rpl16* and *rpoC1*. The Pi values of these regions were calculated in this study, and all but *psbA-trnH* (0.06656) showed a very low Pi of 0.02510 or less (Fig 4 and S2 Table). Therefore, the low phylogenetic resolution of the previous studies was due to the selection of molecular marker regions with very low Pi values.

The results of this study showed that the Pi values of ten noncoding regions (*rps19*-*trnH*, *trnH*-*psbA*, *trnG*-*trnR*, *trnD*-*trnY*, *psbZ*-*trnG*, *petA*-*psbJ*, *petG*-*trnW*, *rpl16*-*rps3*, *rpl2-rpl23*, *and ndhF-trnL*) were relatively high (>0.03). For the selection of useful phylogenetic markers, however, the gene length and PIC also must be considered. Among the ten regions, four regions (*rps19-trnH*, *petG-trnW*, *rpl16-rps3*, *rpl2-rpl23*) are too short to be used as phylogenetic molecular markers. Therefore, that the other six regions will presumably be very useful for resolving the many unclear phylogenetic relationships of the genus *Viola*.

### Phylogenetic implications

The phylogenetic analysis in this study produced an ML tree very similar to that of the Angiosperm Phylogeny Group (APG) system [41]. However, in the APG system, the main clade of Malpighiales was an unresolved polytomy, while in this study, the phylogenetic tree formed a monophyly as follows: Erythroxylaceae and Chrysobalanaceae formed a clade, and Euphorbiaceae formed a sister of the Salicaceae and Violaceae clade. These results are attributed to the increase in resolution resulting from the greater amount of sequence data used in this study. However, only a few species were included in this study, so additional studies that include more species are needed to clarify the phylogenetic relationships in Malpighiales.

In a previous study, the phylogenetic position of *V*. *seoulensis* was not identified, as it formed an unresolved polytomy with *V*. *phalacrocarpa* [11]. Based on the results of the phylogenetic analysis in the present study, it was not possible to confirm the exact phylogenetic position of *V*. *seoulensis* because not enough species were included in the analysis, but *V*. *seoulensis* was the most closely related to *V*. *phalacrocarpa*. An analysis of the chloroplast genomes of the two species in this study revealed that the total genome size of *V*. *phalacrocarpa* was 1335 bp longer than that of *V*. *seoulensis*, and the LSC, IR, and SSC junctions also largely differed between the two species. Additionally, the Pi between the two species was 2.22%. Therefore, it would be reasonable to recognize the two taxa as independent species rather than classifying them as variants, and we will carry out additional studies including allied species of *V*. *phalacrocarpa* and *V*. *seoulensis* to clarify their taxonomic positions.

## Conclusion

We first report of the complete chloroplast genome sequences of four *Viola* species (*V*. *mirabilis*, *V*. *phalacrocarpa*, *V*. *raddeana*, and *V*. *websteri*), and analyzed these data compared to published congeneric species in genus *Viola*. Results of this study, six non-coding regions (*trnH*-*psbA*, *trnG*-*trnR*, *trnD*-*trnY*, *psbZ*-*trnG*, *petA*-*psbJ*, *and ndhF-trnL*) will presumably be very useful for resolving the many unclear phylogenetic relationships of the genus *Viola*. Phylogenetic analyses showed that Malpighiales is divided into five clades at the family level. Also, Violaceae and *Viola* were monophyletic, and was divided into two subclades.

## Supporting information

S1 Fig Comparison of five *Viola* chloroplast genome structure using MAUVE program. (TIF)

S2 Fig Sequence alignment of *ycf15* gene in five *Viola* plastid genomes. (TIF)

S1 Table The GenBank accession numbers of all the 22 chloroplast genomes used for phylogenetic analysis. (DOCX)

S2 Table Eta, Pi value, and PICs of 259 homologous loci. (XLSX)
