# Supplementary figures and images for: The complete chloroplast genome sequences of four Viola species (Violaceae) and comparative analyses with its congeneric species
Source: PLoS One. 2019 Mar 20;14(3):e0214162. doi: 10.1371/journal.pone.0214162 (PMC6426196; doi:10.1371/journal.pone.0214162)

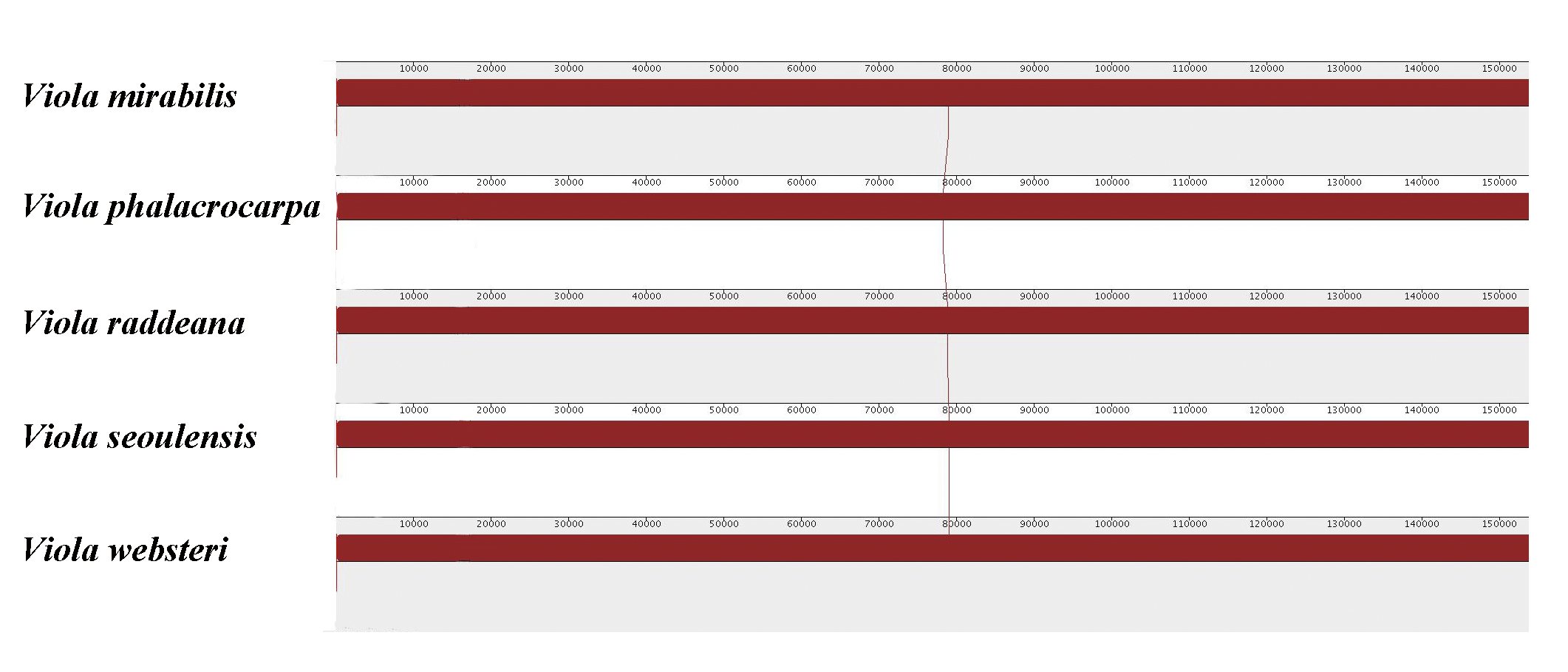

Supplement: S1 Fig — (TIF) [file pone.0214162.s001.tif]

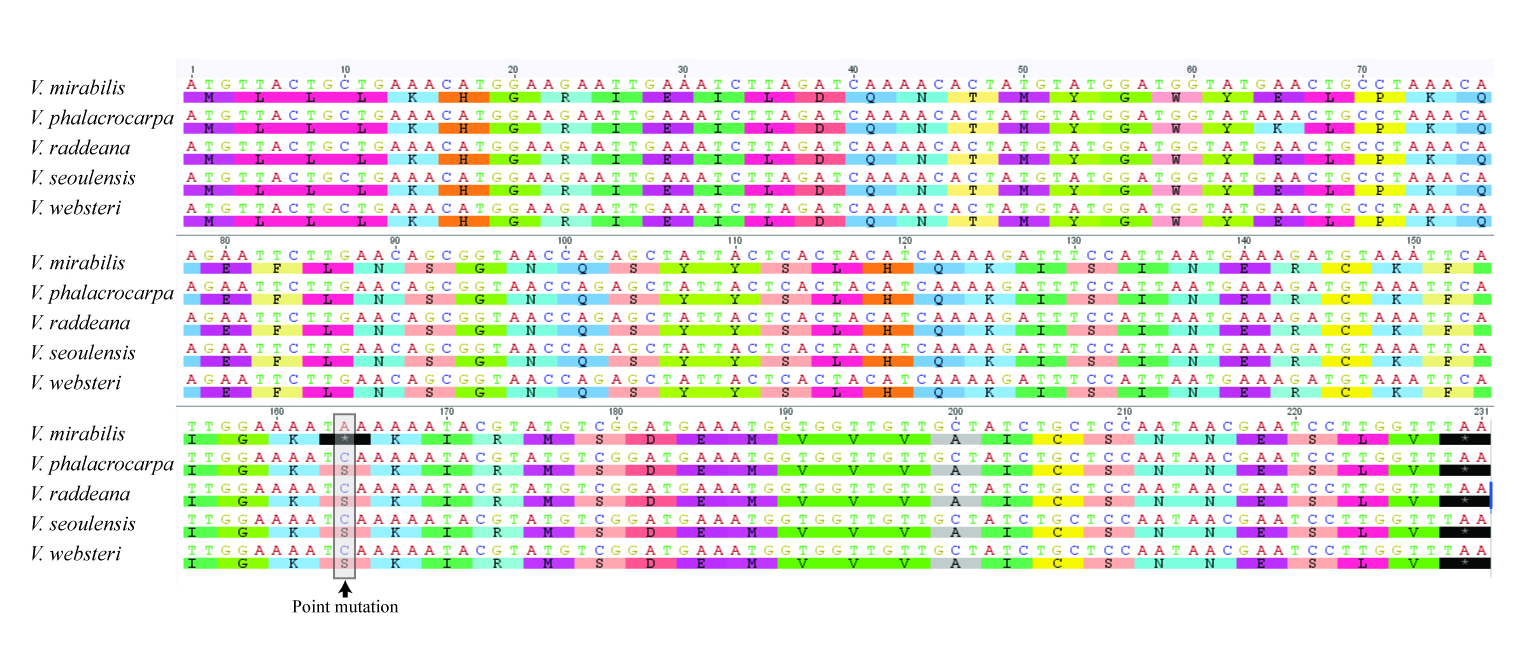

Supplement: S2 Fig — (TIF) [file pone.0214162.s002.tif]
