# Supplementary material for: The complete chloroplast genome sequences of four Viola species (Violaceae) and comparative analyses with its congeneric species
Source: PLoS One. 2019 Mar 20;14(3):e0214162. doi: 10.1371/journal.pone.0214162 (PMC6426196; doi:10.1371/journal.pone.0214162)
Supplement: S1 Table — (DOCX) [file pone.0214162.s003.docx]

**Table S1. The GenBank accession numbers of all the 22 chloroplast genomes used for phylogenetic analysis.**

| Family | Taxon | GenBank Accession No. |
| --- | --- | --- |
| Chrysobalanaceae | *Chrysobalanus icaco* | KJ414480 |
|  | *Couepia guianensis* | KJ414482 |
|  | *Hirtella physophora* | KJ414485 |
|  | *Hirtella racemose* | KJ414479 |
|  | *Licania alba* | KJ414483 |
|  | *Licania heteromorpha* | KJ414481 |
|  | *Licania sprucei* | KJ414484 |
|  | *Parinari campestris* | KJ414486 |
| Erythroxaylaceae | *Erythroxylum novogranatense* | NC030601 |
| Euphorbiaceae | *Hevea brasiliensis* | NC015308 |
|  | *Jatropha curcas* | NC012224 |
|  | *Manihot esculenta* | NC010433 |
|  | *Ricinus communis* | NC016736 |
| Oxalidaceae | *Averrhoa carambola* | KU569488 |
| Salicaceae | *Salix interior* | NC024681 |
|  | *Populus alba* | NC008235 |
|  | *Populus trichocarpa* | NC009143 |
| Violaceae | *Viola mirabilis* | MH229816 |
|  | *Viola phalacrocarpa* | MH229817 |
|  | *Viola raddeana* | MH229818 |
|  | *Viola seoulensis* | KP749924 |
|  | *Viola websteri* | MH229819 |
